# Understanding Patients’ Experiences and Perspectives of Tele-Prehabilitation: A Qualitative Study to Inform Service Design and Delivery

**DOI:** 10.3390/clinpract12040067

**Published:** 2022-08-16

**Authors:** Fiona Wu, Roberto Laza-Cagigas, Tarannum Rampal

**Affiliations:** 1 General Surgery, Darent Valley Hospital, Dartford DA2 8DA, UK; 2 Kent and Medway Prehab, Phase B CIC, London E1 6RA, UK

**Keywords:** cancer, oncology, motivation, aptitude, patient outcome assessment

## Abstract

Background: Tele-prehabilitation is a behaviour change intervention that facilities the modification of unhealthy lifestyle behaviours. Understanding patients’ experiences of tele-prehabilitation provides important insights into service improvement. In this study, we aimed to describe our patients’ perceptions of tele-prehabilitation and capture their capabilities, opportunities, and motivations to participate. This was a qualitative study to inform our service design and delivery. Methods: Following purposive sampling, 22 qualitative semistructured interviews were conducted with patients in the community that had completed tele-prehabilitation. Interviews were recorded and transcribed. Deductive content analysis was used to map the identified themes against theoretical determinants of health behaviour change. Results: We conducted 22 interviews. Our patients described their overall experience of tele-prehabilitation as positive and provided important insights that impacted their capabilities, opportunities, and motivations to engage with our service. Our team provided them the capabilities and self-efficacy to engage by personalising multimodal plans and setting goals. The remote delivery of our service was a recurring positive theme in providing flexibility and widening accessibility to participation. A missed opportunity was the potential for peer support through shared experiences with other patients. Patients showed greater motivation to participate for immediate perioperative benefit compared to long-term health gains. Conclusion: Patients’ experiences and perspectives of tele-prehabilitation can be enhanced by incorporating the findings from this qualitative study into service redesign and delivery. We recommend: (1) applying holistic principles in care and goal-setting, (2) delivering a combination of home-based and in-centre programmes, and (3) engaging with patients at the start of their cancer journey when they are most motivated. In turn, this can result in more effective uptake, improve adherence to interventions, and greater satisfaction.

## 1. Introduction

Unhealthy lifestyle behaviours (e.g., physical inactivity, poor nutrition, smoking, and harmful levels of alcohol consumption) are risk factors for perioperative morbidity and complications [1]. Modifying these risk factors in acute settings can enhance an individual’s functional capacity and, therefore, improve their physical function and reduce their risk of developing postoperative complications [2]. In the longer term, it is important that ongoing health promotion plays a role within the cancer care continuum [3]. The ramifications of cancer treatment can continue to have a detrimental effect on patients’ quality of life, leading to disability and reduced occupational or household productivity [4]. For these reasons, it is important for healthcare professionals and patients to understand that cancer is understood as both an acute illness and one with long-term ill-health consequences.

The time of cancer diagnosis may offer a window of opportunity to encourage patients to change unhealthy lifestyle behaviours when they may be more receptive to change [5]. Behaviour change may only be transient depending on the strength of the motivating factors. Individuals may only change behaviours in the immediate period before surgery for perioperative benefit or make sustained healthier lifestyle changes to better their long-term general health [6,7]. Prehabilitation is a behaviour change intervention for patients to participate in their preoperative care [8,9]. This intervention is aimed at optimising an individual’s physical functionality and psychological wellbeing before surgery to maintain a normal level of function during and after their treatment [10]. Prehabilitation promotes and facilitates healthier lifestyle changes by modifying behavioural patterns [11]. Tele-prehabilitation is an innovative service in delivering prehabilitation interventions in a telecommunication format [12]. Our tele-prehabilitation service was adopted at the start of the COVID-19 pandemic and was expected to provide a sustainable solution to the lockdown restrictions placed during the pandemic [13].

Despite the advertised potential benefits of prehabilitation, not all patients offered our service choose to engage [14,15]. Gaining a better understanding of the actions that underline and influence cancer patients’ behaviours is an important step to developing an effective and evidence-based prehabilitation programme. Guidance from the U.K. Medical Research Council recommends that the development of interventions should be based on evidence and theory [16]. A service that is evidenced in behavioural change theory may result in more effective uptake, improve adherence, and greater satisfaction. The capability, opportunity, motivation, and behaviour (COM-B) model of behaviour change is a theoretical framework that has been used to design and better understand behaviour change interventions [17]. The COM-B model recognises that there are three components to any behaviour change, and all three components must be met for an individual to perform a particular behaviour. The COM-B model proposes that the person (1) needs to have the psychological and physical capability to perform the behaviour, (2) the physical and social opportunity to engage in it, and (3) must be motivated to engage [18,19].

The COM-B model has been successfully applied to a variety of health behaviours [20,21]. Few researchers have investigated patients’ experiences and needs of tele-prehabilitation using the COM-B model [22,23,24]. The present study was conducted to evaluate our service by exploring our patients’ capabilities, opportunities, and motivations to participating in our tele-prehabilitation service. The themes identified from these interviews were mapped to the COM-B model. The COM-B model was chosen over other health behaviour theories because it has the complexity to guide our understanding of a particular behaviour of interest (e.g., participating in prehabilitation). This allows for the identification of the sources of behaviour, and it can be used to identify the barriers and facilitators to behaviour change. Understanding the determinants of behaviour change is important to developing effective interventions. The findings from this study allow us to identify recommendations for service redesign and delivery and to widen participation and improve uptake into our tele-prehabilitation programme. The aim of this study was to qualitatively explore our patients’ experiences and perspective of tele-prehabilitation, which enabled them to engage in tele-prehabilitation, using the COM-B model of behaviour change.

## 2. Materials and Methods

### 2.1. Study Design

We conducted a descriptive qualitative study using individual, semistructured interviews with our past participants. A qualitative research method was chosen as it best captured our patients’ personal accounts of their care. We applied the COM-B model to explore our patients’ experiences and perspectives of tele-prehabilitation to enable us to better understand their behaviours for participating. Individual interviews were chosen over focus groups for two reasons: (1) not all patients would feel comfortable discussing weight management and diet in front of others and (2) rapport can be built in one-to-one interviews encouraging individuals to discuss more sensitive topics. Quantitative data on patient demographics were collected to provide context and to aid the transferability of our qualitative findings.

### 2.2. Participants

This was a follow-up of patients that had enrolled and completed a structured tele-prehabilitation programme from May 2020 to June 2021. This study formed part of our service evaluation into our tele-prehabilitation programme. Eligible patients were: (1) adults (≥18 years) with a new diagnosis of cancer, (2) able to converse in English, (3) had participated in our tele-prehabilitation programme, and (4) were based in the community (i.e., had been discharged from the hospital following surgery). The exclusion criteria included declining to participate in tele-prehabilitation and failure to complete the programme. Patients were recruited from across the southeast region of England from multiple oncological disciplines. Potential interview candidates were identified and invited to participated by a prehabilitation instructor and a consultant in perioperative care.

The sample included 22 patients who were recruited using purposive sampling. Purposive sampling ensures perspectives from a variety of demographics and can produce patterns across the different groups to be identified. A sample size of 22 patients was deemed sufficient to support the data and provide a variety of patient experiences. Furthermore, we determined the sample size when the concept of information power was achieved. Information power is based on the principle that the more information the sample holds; the fewer the participants are needed [25,26]. Thematic saturation is reached when no new themes are generated from the analysis. This was achieved after 22 interviews; therefore, recruitment was ceased.

### 2.3. Ethical Considerations

We consulted the NHS Research Ethics Committee, which stated that no formal approval was required for this study as it was considered a service evaluation. Data were based on the assessment of service delivery, and we were looking to evaluate a community tele-prehabilitation programme that required participants to self-refer. We were evaluating the standard of our service and whether it was meeting the needs of the individuals who chose to participate.

All patients received information about the service evaluation, and they were informed that they could withdraw at any time. Patients provided informed consent to participate in tele-prehabilitation and for their data to be used in the assessment of our service design and delivery. The study was conducted according to the ethical statements of the Declaration of Helsinki.

### 2.4. Prehabilitation

Our prehabilitation service is community-based and delivered through telecommunication methods (termed tele-prehabilitation) [12]. This format has enabled us to provide equitable access across the county. We receive referrals across the southeast region of England from nine acute National Health Service (NHS) Trusts. We provide our patients a multimodal intervention comprising of four key components: (1) a personalised exercise programme, (2) education on nutrition and healthy eating, (3) support and advice on smoking cessation and alcohol reduction, and (4) counselling (including cognitive behavioural therapy). The approach was developed by a core team of professionals, including an anaesthetist, a general practitioner (GP), exercise physiologists, counsellors, public health and wellbeing leads, and public health support staff, with feedback from a patient steering group.

### 2.5. Data Collection

We conducted 30 min semistructured interviews with patients who had completed tele-prehabilitation within 3 to 12 months. Interviews were conducted over the phone or via videoconference call according to patient preference with a member of the research team. The interviewer introduced the service evaluation and confirmed patient consent to participate. The interview questions were developed with input from our multidisciplinary team. These questions involved gathering information on their experiences and perceptions of prehabilitation. An interview guide was used to direct the interview toward specific topics (Table 1). A semistructured interview guide steered discussions to cover the three components of the COM-B model. It consisted of open-ended questions to build an understanding of the capability, opportunity, and motivation components needed to facilitate a behaviour change (i.e., engage with tele-prehabilitation and adhere to our intervention recommendations). The interviewer used prompts and probing as appropriate to elicit more in-depth responses to the questions.

### 2.6. Data Analysis

The interviews were recorded and transcribed verbatim by colleagues from both the public health and research teams, and all data were de-identified. The study was conducted in accordance with the principles of the Declaration of Helsinki. Interview transcriptions were analysed to identify themes and conceptual categories. A deductive approach was taken to explore themes that aligned with the COM-B model.

The Braun and Clarke method for thematic analysis was applied for data analysis [27].The transcribed data were read multiple times to develop familiarity. Data segments that were relevant to the aim of the study were coded. Codes were grouped together to identify patterns and generate themes. Themes were reviewed and defined according to the categories of the COM-B model’s determinants of health behaviour change: capability, opportunity, and motivation. Analysis was manually performed using Microsoft Word (Microsoft Inc., Redmond, WA, USA).

## 3. Results

### 3.1. Patient Characteristics

We invited 22 patients to take part in the interviews, and all agreed to be interviewed. The male-to-female patient ratio was even. The median age of our patient was 66 years (range 42 to 83 years). Most the patients self-identified as White British and had a colorectal cancer diagnosis. Patients beyond the county were able to participate in our programme. Patient demographics and characteristics are outlined in Table 2.

### 3.2. Overview of Themes

Patients provided key insights based on their experiences and perspectives that impacted their capabilities, opportunities, and motivations to participate in tele-prehabilitation (Table 3). The initial coding framework was organised into three categories according to the components of the COM-B model: patients’ (1) capabilities, (2) opportunities, and (3) motivations to participate in our service.

#### 3.2.1. Component 1. Patients’ Capabilities to Participate

##### Theme 1.1. Information and Knowledge

As prehabilitation is relatively new, most of our patients did not have the abilities to participate because they were unaware of the programme before their cancer diagnosis. Patients often did not know what support was locally available to them. Many only heard about the service from other healthcare professionals (e.g., their oncologist, surgeon, or cancer nurse specialist) or word of mouth from other cancer patients promoting our programme when seeking for support after their diagnosis.

*“People want to engage with your kind of service, they just don’t know how to and unfortunately general practitioners (GPs) are so busy, and they don’t have time… The first professional person that mentioned prehabilitation to me was my oncologist… It would be good for our oncologists to initiate (prehabilitation) earlier on…”*

(Woman, 42 years, breast cancer)

They described the interactions with members of the prehabilitation team as key facilitators in their ability to participate. A few patients felt unsupported from their hospital care and sought support from our team. This was particularly welcome during the period when COVID-19 lockdown restrictions were in place and patients were advised to socially distance. The regular interactions served as an opportunity to build a rapport with their prehabilitation professional, lessening feelings of social isolation. Patients spoke positively about their initial encounter with a member of the prehabilitation team. They felt well-informed about the programme. Information was provided in pieces and in a way that was easy to understand and absorb.

*“Having the phone call and explanation from (my prehabilitation professional) helped with my decision.”*

(Man, 49 years, colorectal cancer)

##### Theme 1.2. Personalised Service

Some of the patients were advised to lose weight or quit smoking but did not have the knowledge or abilities to do so. Patients received a variety of resources from the prehabilitation team. They were able to increase their understanding of different exercises and nutrition that would contribute to a quicker recovery. Specifically, our exercise physiologists tailored their advice and plans according to the treatment the patient was receiving, which some of our patients felt was not readily available to them outside of the prehabilitation programme.

*“My (prehabilitation professional) was teaching me exercises after my breast operation… It was really helpful, I couldn’t have got rid of the cording without his advice and guidance, because the physio booklet that I’d been given from the hospital mentioned nothing about cording, it only mentioned to exercise.”*

(Woman, 42 years, breast cancer)

##### Theme 1.3. Multimodal Programme

A key theme highlighted during the interviews was the multimodal approach to the prehabilitation programme. Many perceived that their abilities to adhere to the interventions required a holistic approach. The counselling offered as part of the service was a key component to psychologically prepare the patients to engage in the service.

*“I see them as all together. Without the mental part, it is difficult to have the motivation to keep doing the exercises and keep the nutrition side of things going. It’s the mental part that really is important. The nutrition helps the brain to be able to be in the right mindset and helps give you that energy. So, I see the three as being very much all together as part of a triangle. If you take one away, it doesn’t work.”*

(Man, 49 years, colorectal cancer)

##### Theme 1.4. Self-Efficacy

The prehabilitation programme played a role in improving the individuals’ self-efficacy. We applied a holistic approach to the risk factor screening assessment at their initial tele-prehabilitation session. The exercise physiologist collaborated with each individual patient to design a personalised plan and set goals. Achieving these goals brought them feelings of success and empowerment (i.e., the more goals they were able to achieve, the more confident they became in achieving those goals). This enabled them to develop the necessary skills and psychological capabilities to manage their own health.

*“Now I have a feeling of control over my body… I don’t want cancer to define me.”*

(Woman, 48 years, colorectal cancer)

#### 3.2.2. Component 2. Patients’ Opportunities to Participate

##### Theme 2.1. Convenience of Tele-Prehabilitation

A prominent theme highlighted during the interviews was the convenience of tele-prehabilitation. Patients appreciated that tele-prehabilitation provided an alternative solution during the COVID-19 lockdown restrictions. For some patients, the remote delivery of a home-based programme provided a degree of flexibility and convenience to enable them to incorporate exercise into their daily routines and around their work or personal commitments. In addition, they agreed that some individuals may not have access to hospital transport (e.g., prohibited by the cost of public transport or hospital parking) or had time constraints.

##### Theme 2.2. Widening Accessibility

The patients appreciated that the tele-prehabilitation programme has the potential to widen accessibility. Some patients mentioned that the provision of telehealth allowed them to access the service when, typically, it would geographically fall outside of their catchment area. Furthermore, a few patients would have been unable to travel to in-centre appointments because of their physical limitations. Their physical symptoms (e.g., pain, poor mobility, or diarrhoea) would have restricted them from participating in the in-centre supervised sessions.

*“Having prehabilitation outside of the hospital setting made things easier. I wasn’t feeling good with the pain and couldn’t travel too far. Could also do it in my own time.”*

(Man, 66 years, did not disclose diagnosis)

Moreover, a few patients with psychological issues (e.g., anxiety or overthinking) would have found it difficult to attend prehabilitation sessions in a hospital setting. They mentioned that hospital visits and clinical interactions with hospital staff heightened their levels of anxiety and created stress as they were associated with receiving bad news and it would have been a barrier for them to attend in-centre prehabilitation.

*“Hospital appointments were associated with days with low mood… Never quite sure what the hospital agenda is.”*

(Man, 62 years, colorectal cancer)

##### Theme 2.3. Community

A key opportunity patients felt was missing from tele-prehabilitation was the chance to share advice with other patients. A few patients expressed feelings of isolation and would often seek advice and support from online patient forums. They were concerned about being a burden on their family and wanted to spare them feelings of fear and worry. They would have liked to have met other cancer patients with similar experiences. They felt that the in-centre sessions would have provided access to other patients to create a community, enabling them to share their experiences with each other and provide peer support. They believed in-centre prehabilitation can result in a higher success rate owing to the support from patients with shared experiences.

*“I would have liked to have contact with other people that are going through cancer or have had gone through cancer… You don’t want to burden your friends or your family with what you’re going through. So perhaps having like a face-to-face support group might have been helpful or perhaps group sessions.”*

(Woman, 49 years, colorectal cancer)

##### Theme 2.4. Postdischarge Support

Patients valued the ongoing support from the prehabilitation team on discharge. The access to the services on discharge, particularly during difficult periods of their recovery, was an opportunity to embed positive changes.

*“I would talk to him about the issues I was having, the first couple of weeks it was more about recovery fatigue… He was checking that I was drinking enough, I was eating the right foods, and lots of the stuff that I knew, but it’s nice to have someone reinforce that.”*

(Woman, 42 years, breast cancer)

#### 3.2.3. Component 3. Patients’ Motivations to Participate

##### Theme 3.1. Health Outcomes

A prominent theme was the emotional response to receiving a cancer diagnosis. Patients were motivated to modify their lifestyle to optimise their health as a result of this new diagnosis. In acute settings, all the patients believed that participation in our service would enable them to withstand the adverse consequences of their cancer treatment, leading to better health outcomes and quicker recovery. A few patients felt concerned about their health given the diagnosis and were motivated to change to beat their disease. Some perceived prehabilitation to be a core component of their cancer treatment. Others reported general long-term health-related reasons as a motivation to adopt a healthier lifestyle, including reducing the risk of chronic diseases (e.g., cardiovascular disease and diabetes) and improving their quality of life.

*“* *You’ve got to keep your body physically fit… So, if you do have to have aggressive treatment or surgery, then you’re more likely to recover from it.”*

(Woman, 66 years, did not disclose diagnosis)

##### Theme 3.2. Influence of Friends and Family

Our patients agreed that the support from their family and friends served as a strong motivator of their engagement in the prehabilitation programme. They frequently described how other household members would also engage in the programme and provide support and encouragement, which aided behavioural changes.

*“Occasionally, I will take my youngest to a fast-food restaurant but it’s not such a regular treat anymore… It’s about the generation change, I want him to be more active and build all those things into our lifestyle.”*

(Woman, 49 years, colorectal cancer)

##### Theme 3.3. Patient–Professional Relationship

Patients said the availability of support from the prehabilitation team helped to track their progress and maintain engagement. Patients had regular one-to-one interactions with a member of the prehabilitation team, enabling them to build trust and rapport. The weekly or biweekly phone calls served as an opportunity to set goals, and encourage and reinforce healthy behaviours. The positive interactions between the patient and their prehabilitation professional facilitated healthier behaviour changes.

*“I had one-to-one contact with (prehabilitation professional), so I was able to speak to him and kind of build up a rapport… As with the counsellor, I think having been able to have that continuity with the support I had was very important to me. I found that quite a challenge for me to reach out to accept help or to ask for help.”*

(Woman, 42 years, breast cancer)

The prehabilitation team emphasised the use of monitoring, plans and goals. Many patients often monitored their exercises and diet and set their own goals. They were motivated to achieve their goals to return to their normal routines after finishing their cancer treatment.

*“I’ve gone through a week of intense radiotherapy, 18 weeks of chemo and surgery. I’m walking the kids to school. I’m taking the dog for a walk. I’m going to the gym. Let’s see how you get on.”*

(Man, 49 years, colorectal cancer)

##### Theme 3.4. Positive Mindset

Many patients had a positive mindset toward their health outcomes and disease. They perceived this positive mindset as a motivation to engage with our intervention programme and to cope with their disease and recovery.

*“* *The advice that I had from (prehabilitation professional), and the counselling I got, certainly put me in a more positive mental attitude, a positive emotional attitude, but also helped me physically to be in the right place as well.”*

(Man, 62 years, colorectal cancer)

## 4. Discussion

In this study, we sought to understand the experiences and perspectives of our tele-prehabilitation programme amongst a diverse group of patients living with cancer as part of our service evaluation. Understanding these is an important element of a patient-centred approach as they will influence their decision to engage with our service. The diagnosis of cancer is a life-changing event that offers a teachable moment and an opportunity for behaviour change [28]. These behaviour changes may only be transient in the absence of a support programme. It is important that our tele-prehabilitation service is modelled on theories of behavioural change to enhance uptake. Here, we provide several key recommendations for service redesign and delivery based on our findings.

Our study provides the insight that prehabilitation can promote an element of self-efficacy. Self-efficacy is an important concept: it refers to an individual’s belief to whether they can perform a challenging task under specific circumstances [29]. At the start of the intervention, our exercise physiologists collaborated with each patient to establish personalised goals. Achieving these goals produced feelings of success and empowerment in the patients. In turn, this developed their confidence to believe they had the physical and psychological capabilities to continue making positive behaviour changes. A study looking at oesophageal cancer patients found a correlation between self-efficacy and better health outcomes and improved quality of life [30]. Prehabilitation must promote self-efficacy for this reason. We recommend the following aspects to consider when designing a prehabilitation service: (1) the plans need to be personalised, (2) patients should be taught the necessary skills to self-manage their health, and (3) the goal should be appropriate and attainable according to each patient.

A prominent theme extracted from the analysis was the convenience and accessibility of our tele-prehabilitation service. This approach to delivering prehabilitation can address some of the recruitment issues highlighted in previous studies [14,15]. Before COVID-19, our patients had to rely on travelling to the hospital for scheduled prehabilitation appointments. The studies examining the feasibility of in-centre prehabilitation showed that the commonest barriers to engagement are occupational or household commitments and patient unwillingness, or inability, to travel to the hospital. Patients welcomed the flexibility of our tele-prehabilitation service adaptation in this study. Patients did not need to travel, enabling those with a lack of access to hospital transport or those living outside of the region to participate. Patients were able to engage in our intervention in the safety and comfort of their own home, which may have facilitated engagement for some patients with physical limitations or those suffering with hospital anxiety. The inflexibility of in-centre prehabilitation, due to the fixed timing and locations, may limit recruitment and service uptake. Studies examining the adherence of exercise training interventions during chemotherapy amongst breast cancer patients concluded that convenience is a positive predictor for adherence [31,32]. Furthermore, a review looking at the cost-effectiveness of telemedicine showed that this method of delivering healthcare has the potential to reduce costs [33]. Tele-prehabilitation can provide cost-saving benefits to patients (e.g., costs for fuel or hospital parking, childcare, and gym memberships). The financial cost of attending in-centre prehabilitation may be a barrier for engagement for some patients. For these reasons, tele-prehabilitation can play a role in widening participation and provide the opportunity for patients who may have previously declined to take part in prehabilitation to engage.

A disadvantage of tele-prehabilitation is the missed opportunity for peer support. Our tele-prehabilitation patients often sought support and advice from other cancer patients through online patient forums. Patients felt in-centre prehabilitation would provide opportunities to meet other patients, enabling them to share their experiences and to give or receive advice. Oesophageal cancer patients found peer support was important to minimising feelings of isolation and providing coping advice that could not be provided by friends or family [34,35]. Bringing patients together to discuss shared experiences can create a sense of shared identity and social belonging [36,37]. Peer support programmes can provide both educational and emotional benefits [38]. It is imperative that we incorporate these findings to improve the quality of our service to produce better patient outcomes and experiences for future participants. We envisage that the redesign of our service will include a combination of remote delivery for the convenience and in-centre sessions to allow patients to come together to share their own lived-in experiences. In addition, we anticipate that there will be a cohort of patients that will prefer to meet other patients remotely; therefore, we have created an online peer support forum for patients to discuss their own experiences.

Our patients identified several motivations for engaging in prehabilitation, including cancer outcomes, support from friends and family, and the rapport developed with their prehabilitation professional. A recurring theme motivating patients to participate in prehabilitation was their ability to change the course of their recovery. Patients believed prehabilitation would enable them to withstand the consequences of their treatment and recover quicker. All our patients reported a favourable attitude toward participating in prehabilitation to change their health behaviours for immediate perioperative benefit. Our finding is in keeping with that of a study that explored patients’ attitudes toward behaviour change interventions. Their cohort of patients were more motivated to change for perioperative benefit than for long-term health gains [7,39]. Prehabilitation can be a teachable moment to positively influence patients to change their unhealthy lifestyle behaviours [28]. We recommend that prehabilitation is introduced at the start of the patients’ cancer diagnoses; patients are more likely to be motivated to participate, and in turn, this can maximise their health outcomes.

The application of the COM-B model provided a starting point to better understand the barriers and facilitators to participating in tele-prehabilitation. It highlights three distinct explanatory components and the potential influences on behaviour change. We found that our patients’ experiences with tele-prehabilitation were mostly positive, which in turn impacted their capabilities, opportunities, and motivations to participate. We provided several recommendations for service improvement from this study. Furthermore, we recommend incorporating prehabilitation within Public Health England’s strategy for people with newly diagnosed cancer and cancer survivors [40]. The established integrated care systems may help contribute toward the United Kingdom’s Cancer Prevention Strategy.

### Limitations

We aimed to recruit a sample who we deemed would be most representative of the patients that would participate in our programme with our purposive sampling strategy. However, this nonprobability sampling had the potential to introduce selection bias. The age range of our study population was wide; our interviewees ranged from 42 to 83 years old. This was reflected in our study population, who were older by default, because the incidence rates for all cancers combined are highest amongst the older population [41]. In 2016–2018, more than one-third of new cancer cases were diagnosed in people aged 75 year and over in the United Kingdom. The highest incidence rates were observed in those aged between 85 and 89 years old.

The patients self-selected to participate in the programme, which suggests that they may have already had an element of self-efficacy and motivation to participate in behaviour change interventions. Patients’ responses were dependent on their personal recall of their own experiences, which may not be representative of our population or generalisable to other settings. The nature of the questions may have influenced the answers provided. We had a small sample size. A consideration in future studies may be to interview patients who declined to engage with our service. Understanding their experiences and perceptions for nonparticipation would add value to our service evaluation and provide a broader perspective for intervention development. Furthermore, there was an element of observer bias.

## 5. Conclusions

Our findings provided insight into the factors important for designing behaviour change intervention services. Patients’ positive experiences with tele-prehabilitation impacted their capabilities, opportunities, and motivations to participate. Understanding behaviour change theories and applying them in practice has the potential to yield effective interventions.

## Figures and Tables

**Table 1:** Interview guide used to facilitate the interviews.

Component of the COM-B Model	Questions	Prompts/Probes
Capability	How were you able to take part in tele-prehabilitation?	Are there any barriers or facilitators to joining tele-prehabilitation?
	Which part of the programme did you find most beneficial?	What skills have you learnt? Which skills have you been able to use?
Opportunity	What are your experiences of virtual prehabilitation?	Are there any benefits or disadvantages of delivering prehabilitation remotely? Do you have any recommendations for future delivery?
Motivation	Why did you take part in prehabilitation?	What motivated you to be in better health?

**Table 2:** Summary of patients’ characteristics (n = 22).

Characteristics of Patients	*n*	%
Sex		
Male	11	50.0%
Female	11	50.0%
Ethnicity		
White: English, Welsh, Scottish, Northern Irish, Irish, British, or any other White background	19	86.4%
Black: African, Caribbean, or Black British	2	9.1%
Did not disclose	1	4.5%
Cancer origin		
Colorectal	17	77.3%
Breast	2	9.1%
Urology	1	4.5%
Did not disclose	2	9.1%
NHS Trust providing care		
Lewisham and Greenwich NHS Trust	7	31.8%
Medway NHS Foundation Trust	6	27.3%
Maidstone and Tunbridge Wells NHS Trust	6	27.3%
Oxford University Hospitals NHS Foundation Trust	1	4.5%
Gloucestershire Hospitals NHS Foundation Trust	1	4.5%
Did not disclose	1	4.5%

**Table 3:** Emerging themes generated from our analysis.

Component of the COM-B Model	Emerging Themes	Concepts
Capability	Information and knowledge	Awareness of programme Assistance in health promotion
	Personalised service	Tailored advice according to patients’ treatments and symptoms
	Multi-modal programme	Holistic approach to improving one’s health
	Self-efficacy	Having skills to adopt healthier behaviours Sense of achievement and self-worth
Opportunity	Convenience	Flexibility around schedule
	Widening accessibility	Access to service outside of catchment area Comfort and safety of own home Hospital anxiety
	Community	Lack of shared patient experiences and peer support
	Post-discharge support	Ongoing access to support services on discharge
Motivation	Health outcomes	Enhanced recovery Overcoming their disease
	Influence of friends and family	Support from friends and family
	Patient–professional relationship	Regular communication and encouragement from prehabilitation professionals Creating plans and goal setting to promote healthier behaviours Positive reinforcement
	Positive mindset	Positive outlook to beat their disease

## Data Availability

The data presented in this study are available on request from the corresponding author. Data are not publicly available due to privacy concerns.

## References

[1] Van Wijk L., van der Snee L., Buis C.I., Hentzen J.E.K.R., Haveman M.E., Klaase J.M. (2021). A prospective cohort study evaluating screening and assessment of six modifiable risk factors in HPB cancer patients and compliance to recommended prehabilitation interventions. Perioper. Med..

[2] Durrand J., Singh S.J., Danjoux G. (2019). Prehabilitation. Clin. Med..

[3] Mina D.S., Van Rooijen S.J., Minnella E.M., Alibhai S.M.H., Brahmbhatt P., Dalton S.O., Gillis C., Grocott M.P.W., Howell D., Randall I.M. (2021). Multiphasic Prehabilitation Across the Cancer Continuum: A Narrative Review and Conceptual Framework. Front. Oncol..

[4] Catto J.W., Downing A., Mason S., Wright P., Absolom K., Bottomley S., Hounsome L., Hussain S., Varughese M., Raw C. (2021). Quality of Life After Bladder Cancer: A Cross-sectional Survey of Patient-reported Outcomes. Eur. Urol..

[5] Sosnowski R., Kamecki H., Bjurlin M.A., Przewoźniak K. (2019). The diagnosis of bladder cancer: Are we missing a teachable moment for smoking cessation?. Transl. Androl. Urol..

[6] Yang C.C., Liu C.Y., Wang K.Y., Chang Y.K., Wen F.H., Lee Y.C., Chen M.L. (2021). Trajectory of smoking behaviour during the first 6 months after diagnosis of lung cancer: A study from Taiwan. J. Adv. Nurs..

[7] Hirschey R., Nyrop K.A., Mayer D.K. (2020). Healthy Behaviors: Prevalence of Uptake Among Cancer Survivors. Clin. J. Oncol. Nurs..

[8] Minnella E.M., Awasthi R., Loiselle S.E., Agnihotram R.V., Ferri L.E., Carli F. (2018). Effect of Exercise and Nutrition Prehabilitation on Functional Capacity in Esophagogastric Cancer Surgery: A Randomized Clinical Trial. JAMA Surg..

[9] Carli F., Silver J.K., Feldman L.S., McKee A., Gilman S., Gillis C., Scheede-Bergdahl C., Gamsa A., Stout N., Hirsch B. (2017). Surgical Prehabilitation in Patients with Cancer: State-of-the-Science and Recommendations for Future Research from a Panel of Subject Matter Experts. Phys. Med. Rehabil. Clin. N. Am..

[10] Santa Mina D., Scheede-Bergdahl C., Gillis C., Carli F. (2015). Optimization of surgical outcomes with prehabilitation. Appl. Physiol. Nutr. Metab..

[11] Tew G.A., Bedford R., Carr E., Durrand J.W., Gray J., Hackett R., Lloyd S., Peacock S., Taylor S., Yates D. (2020). Community-based prehabilitation before elective major surgery: The PREP-WELL quality improvement project. BMJ Open Qual..

[12] Waterland J.L., Chahal R., Ismail H., Sinton C., Riedel B., Francis J.J., Denehy L. (2021). Implementing a telehealth prehabilitation education session for patients preparing for major cancer surgery. BMC Health Serv. Res..

[13] Wu F., Rotimi O., Laza-Cagigas R., Rampal T. (2021). The Feasibility and Effects of a Telehealth-Delivered Home-Based Prehabilitation Program for Cancer Patients during the Pandemic. Curr. Oncol..

[14] Wu F., Laza-Cagigas R., Pagarkar A., Olaoke A., El Gammal M., Rampal T. (2021). The Feasibility of Prehabilitation as Part of the Breast Cancer Treatment Pathway. PM&R.

[15] Martin D., Besson C., Pache B., Michel A., Geinoz S., Gremeaux-Bader V., Larcinese A., Benaim C., Kayser B., Demartines N. (2021). Feasibility of a prehabilitation program before major abdominal surgery: A pilot prospective study. J. Int. Med. Res..

[16] Craig P., Dieppe P., Macintyre S., Michie S., Nazareth I., Petticrew M. (2008). Medical Research Council Guidance. Developing and evaluating complex interventions: The new Medical Research Council guidance. BMJ.

[17] Mersha A.G., Gould G.S., Bovill M., Eftekhari P. (2020). Barriers and Facilitators of Adherence to Nicotine Replacement Therapy: A Systematic Review and Analysis Using the Capability, Opportunity, Motivation, and Behaviour (COM-B) Model. Int. J. Environ. Res. Public Health.

[18] Keyworth C., Epton T., Goldthorpe J., Calam R., Armitage C.J. (2020). Acceptability, reliability, and validity of a brief measure of capabilities, opportunities, and motivations (“COM-B”). Br. J. Health Psychol..

[19] Michie S., van Stralen M.M., West R. (2011). The behaviour change wheel: A new method for characterising and designing behaviour change interventions. Implement. Sci..

[20] Boyd J., McMillan B., Easton K., Delaney B., Mitchell C. (2020). Utility of the COM-B model in identifying facilitators and barriers to maintaining a healthy postnatal lifestyle following a diagnosis of gestational diabetes: A qualitative study. BMJ Open.

[21] Flannery C., McHugh S., Anaba A.E., Clifford E., O’Riordan M., Kenny L.C., McAuliffe F.M., Kearney P.M., Byrne M. (2018). Enablers and barriers to physical activity in overweight and obese pregnant women: An analysis informed by the theoretical domains framework and COM-B model. BMC Pregnancy Childbirth.

[22] Gillis C., Gill M., Gramlich L., Culos-Reed S.N., Nelson G., Ljungqvist O., Carli F., Fenton T. (2021). Patients’ perspectives of prehabilitation as an extension of Enhanced Recovery After Surgery protocols. Can. J. Surg..

[23] Seery T., Wu F., Hendricks E., McDonald J., Laza-Cagigas R., Elliott S., Rampal T. (2022). Patients’ experiences of virtual prehabilitation during the COVID-19 pandemic. Patient Educ. Couns..

[24] Waterland J.L., Ismail H., Amin B., Granger C.L., Denehy L., Riedel B. (2021). Patient acceptance of prehabilitation for major surgery: An exploratory survey. Support Care Cancer.

[25] Malterud K., Siersma V.D., Guassora A.D. (2016). Sample Size in Qualitative Interview Studies: Guided by Information Power. Qual. Health Res..

[26] Weller S.C., Vickers B., Bernard H.R., Blackburn A.M., Borgatti S., Gravlee C.C., Johnson J.C. (2018). Open-ended interview questions and saturation. PLoS ONE.

[27] Braun V., Clark V. (2006). Using thematic analysis in psychology. Qual. Res. Psychol..

[28] Flocke S.A., Clark E., Antognoli E., Mason M.J., Lawson P.J., Smith S., Cohen D.J. (2014). Teachable moments for health behavior change and intermediate patient outcomes. Patient Educ. Couns..

[29] Holloway A., Watson H.E. (2002). Role of self-efficacy and behaviour change. Int. J. Nurs. Pract..

[30] Magon A., Caruso R., Sironi A., Mirabella S., Dellafiore F., Arrigoni C., Bonavina L. (2021). Trajectories of Health-Related Quality of Life, Health Literacy, and Self-Efficacy in Curatively-Treated Patients with Esophageal Cancer: A Longitudinal Single-Center Study in Italy. J. Patient Exp..

[31] Courneya K.S., Segal R.J., Gelmon K., Mackey J.R., Friedenreich C.M., Yasui Y., Reid R.D., Proulx C., Trinh L., Dolan L.B. (2014). Predictors of adherence to different types and doses of supervised exercise during breast cancer chemotherapy. Int. J. Behav. Nutr. Phys. Act..

[32] Ormel H.L., van der Schoot G., Sluiter W.J., Jalving M., Gietema J.A., Walenkamp A. (2018). Predictors of adherence to exercise interventions during and after cancer treatment: A systematic review. Psycho-oncology.

[33] De la Torre-Díez I., López-Coronado M., Vaca C., Aguado J.S., de Castro C. (2015). Cost-utility and cost-effectiveness studies of telemedicine, electronic, and mobile health systems in the literature: A systematic review. Telemed. J. e-Health.

[34] Graham-Wisener L., Dempster M. (2017). Peer advice giving from posttreatment to newly diagnosed esophageal cancer patients. Dis. Esophagus.

[35] Hoey L.M., Ieropoli S.C., White V.M., Jefford M. (2008). Systematic review of peer-support programs for people with cancer. Patient Educ. Couns..

[36] Dakof G.A., Taylor S.E. (1990). Victims’ perceptions of social support: What is helpful from whom?. J. Personal. Soc. Psychol..

[37] Clarke C., McCorry N.K., Dempster M. (2011). The role of identity in adjustment among survivors of oesophageal cancer. J. Health Psychol..

[38] Mirrielees J.A., Breckheimer K.R., White T.A., Denure D.A., Schroeder M.M., Gaines M.E., Wilke L.G., Tevaarwerk A.J. (2017). Breast Cancer Survivor Advocacy at a University Hospital: Development of a Peer Support Program with Evaluation by Patients, Advocates, and Clinicians. J. Cancer Educ..

[39] McDonald S., Yates D., Durrand J.W., Kothmann E., Sniehotta F.F., Habgood A., Colling K., Hollingsworth A., Danjoux G. (2019). Exploring patient attitudes to behaviour change before surgery to reduce peri-operative risk: Preferences for short- vs. long-term behaviour change. Anaesthesia.

[40] LongTermPlan.nhs.uk. The NHS Long Term Plan. https://www.longtermplan.nhs.uk/wp-content/uploads/2019/08/nhs-long-term-plan-version-1.2.pdf.

[41] Cancer Research UK (2022). Cancer Incidence by Age. https://www.cancerresearchuk.org/health-professional/cancer-statistics/incidence/age#ref-.

